# Comparative genomics of Lactobacillaceae from the gut of honey bees, *Apis mellifera*, from the Eastern United States

**DOI:** 10.1093/g3journal/jkac286

**Published:** 2022-11-04

**Authors:** Emma L Bradford, Noah Wax, Emma K Bueren, Jenifer B Walke, Richard Fell, Lisa K Belden, David C Haak

**Affiliations:** Department of Biological Sciences, Virginia Tech, Blacksburg, VA 24061, USA; Department of Biological Sciences, Virginia Tech, Blacksburg, VA 24061, USA; Department of Biological Sciences, Virginia Tech, Blacksburg, VA 24061, USA; Department of Biology, Eastern Washington University, Cheney, WA 99004, USA; Department of Entomology, Virginia Tech, Blacksburg, VA 24061, USA; Department of Biological Sciences, Virginia Tech, Blacksburg, VA 24061, USA; School of Plant and Environmental Sciences, Virginia Tech, Blacksburg, VA 24061, USA

**Keywords:** *Lactobacillus*, *Apilactobacillus*, *Bombilactobacillus*, whole genome, honey bee microbiome, gut microbiome, prophage, bacteriophage

## Abstract

Lactobacillaceae are an important family of lactic acid bacteria that play key roles in the gut microbiome of many animal species. In the honey bee (*Apis mellifera*) gut microbiome, many species of Lactobacillaceae are found, and there is functionally important strain-level variation in the bacteria. In this study, we completed whole-genome sequencing of 3 unique Lactobacillaceae isolates collected from hives in Virginia, USA. Using 107 genomes of known bee-associated Lactobacillaceae and *Limosilactobacillus reuteri* as an outgroup, the phylogenetics of the 3 isolates was assessed, and these isolates were identified as novel strains of *Apilactobacillus kunkeei*, *Lactobacillus kullabergensis*, and *Bombilactobacillus mellis*. Genome rearrangements, conserved orthologous genes (COG) categories and potential prophage regions were identified across the 3 novel strains. The new *A. kunkeei* strain was enriched in genes related to replication, recombination and repair, the *L. kullabergensis* strain was enriched for carbohydrate transport, and the *B. mellis* strain was enriched in transcription or transcriptional regulation and in some genes with unknown functions. Prophage regions were identified in the *A. kunkeei* and *L*. *kullabergensis* isolates. These new bee-associated strains add to our growing knowledge of the honey bee gut microbiome, and to Lactobacillaceae genomics more broadly.

## Introduction

In recent years, honey bees have emerged as an important system for understanding the functional roles of bacteria in the gut microbiome. Relative to vertebrates, the gut microbiome of honey bees is simplified, with 8–10 dominant bacterial phylotypes (Moran 2015), and important strain-level variation (Engel *et al.* 2014; Ellegaard and Engel 2016; Ellegaard *et al.* 2019). Lactobacillaceae (phylum: Firmicutes, class: Bacilli, order: Lactobacillales) is a family of lactic acid bacteria found within the gut of both vertebrates and invertebrates (Makarova *et al.* 2006; Zheng *et al.* 2020). Lactobacillaceae taxonomy was recently updated, with all genera in the family Leuconostoceae incorporated into Lactobacilliceae, and the predominant genus, *Lactobacillus*, split into 25 genera representing distinct clades (Zheng *et al.* 2020). Genome sequences of new Lactobacillaceae species and strain variants are regularly being published (Kwong *et al.* 2014; Olofsson *et al.* 2014), and these species can vary widely in genome size (from 1,700,000 to ≥3,000,000 base pairs) and GC content (from 31% to 56%) (Felis and Dellaglio 2007; Kant *et al.* 2011). The recent taxonomic changes (Zheng *et al.* 2020) resulted in some honey bee *Lactobacillus* species reclassified as *Apilactobacillus* (with subsequent changes to their specific epithets), and some bumble bee species reclassified as *Bombilactobacillus*. As for many bacterial species, Lactobacillaceae genomes are composed of both species specific genes and additional genetic elements, including bacteriophages (Medini *et al.* 2005), that can result in larger genomes than expected. Temperate bacteriophages have stable relationships with their bacterial hosts and are incorporated into the host genome as prophages (Casjens 2003). A recent study of 1,472 Lactobacillaceae genomes found that 99.8% of the genomes contained predicted prophage regions (Pei *et al.* 2021).

In the honey bee microbiome, Lactobacillaceae phylotypes previously comprised 2 clades: Firm-4 and Firm-5. Firm-5 was reclassified to the *Lactobacillus melliventris* clade (Zheng *et al.* 2020), and are the most common of the honey bee bacterial phylotypes, although other *Lactobacillus* species are also found in high abundance in honey bees (Corby-Harris *et al.* 2014; Anderson *et al.* 2016; Ellegaard and Engel 2019). There is variation in microbiome composition along the honey bee gut, from crop to midgut to hindgut (Powell *et al.* 2014). The crop may be dominated by species that favor acidic, sugar-rich environments, such as *Apilactobacillus kunkeei* (Corby-Harris *et al.* 2014). The midgut contains a small bacterial community, dominated by the Gamma-1 phylotype (Martinson *et al.* 2012). Most bacteria reside in the hindgut, which can be divided into the ileum and the rectum. The ileum contains mainly Gram-negative species, such as *Frischella perrara*, *Snodgrassella alvi*, *Gilliamella apicola*, and some species of the *L. melliventris clade*, while the rectum contains large populations of Gram-positive species, including species in the *L. melliventris* clade, other *Lactobacillus* species, and *Bifidobacterium* species (Martinson *et al.* 2012; Powell *et al.* 2014; Kwong and Moran 2016).

Here, we present 3 new Lactobacillaceae genomes from isolates obtained from honey bees in the Eastern US, and compare them to existing honey bee Lactobacillaceae genomes, which have predominantly been collected in Europe.

## Materials and methods

### Honey bee collection and bacterial isolation

Honey bees were collected from hives at the Virginia Tech apiary in Montgomery County, VA, USA in August (*N* = 1 hive) and September (*N* = 6 hives), 2016. Bees were collected from inside the hive in sterile 50 ml centrifuge tubes and placed on ice until they were returned to the laboratory, where they were frozen at −80°C until they could be dissected.

In the laboratory, bees were briefly thawed and then surface-sterilized using 5% bleach followed by 3 rinses in sterile water (Engel *et al.* 2013). For each individual bee (*N* = 10), the whole gut was dissected using sterile technique, the mid- and hind-guts were separated, and the separate gut regions were placed into 1.5 ml microcentrifuge tubes, each containing 500 μl of sterile 10 mM MgSO_4_ (Kwong and Moran 2013). Sterile pestles were used to homogenize the gut samples, which were serially diluted in 10 mM MgSO_4_ (10^−1^ to 10^−5^), and 200 μl of each dilution was plated onto de Man Rogosa Sharpe (MRS) agar plates. Plates were incubated at 37°C for 2–3 days under low oxygen culture conditions using incubation chambers (BD GasPak™ EZ Gas Generating Systems Incubation Containers) and GasPak™ sachets (BD Bioscience’s GasPak™ Sachets). Morphologically distinct bacterial colonies were further isolated to obtain pure cultures; this was done by visual inspection of colony morphology to ensure pure colonies from between 1 and 3 subcultures.

For each of these isolates, we extracted DNA using the MoBio Laboratories UltraClean Microbial DNA Isolation kit or the Qiagen DNeasy Blood & Tissue kit (Qiagen Inc., Valencia, CA), then amplified and sequenced the full-length 16S rRNA region using primers 8F (5′-AGAGTTTGATCCTGGCTCAG-3′) and 1492R (5′-GGTTACCTTGTTACGACTT-3′), as described in Lauer *et al.* (2008). Isolate PCR products were cleaned using the Qiagen QIAquick PCR Purification Kit and eluted in 50 μl of molecular water prior to Sanger sequencing for assignment to genus. Sequences were determined to be different species based on taxonomic differences from BLAST analysis. Within the set of 27 sequenced isolates, we obtained 17 *Lactobacillus* isolates, and of these, we chose 3 that appeared to be separate species based on the full-length 16S *rRNA* gene sequence for whole-genome sequencing. To extract DNA for whole-genome sequencing, isolates were regrown in 750 μl of MRS broth, shaking at 150 rpm at room temperature for 24 h. The cultures were then centrifuged at 7,500 rpm for 10 min, and the resulting pellet was resuspended in 180 μl of lysis buffer containing 20 mg/ml lysozyme and processed through the Gram-positive bacteria protocol of the Qiagen DNeasy Blood and Tissue Kit, with final elution in 150 μl molecular water.

### Genome assembly

Extracted DNA was sent to the Duke Center for Genomic and Computational Biology for library construction and sequencing on the Illumina Hi-Seq 4000 platform, 2 × 150 bp. This generated an average of 4 Gbp of reads per sample library with an average *Q* score of 34% and 96% of reads >Q30. Raw reads were adapter trimmed using Trimmomatic v.0.35 (Bolger *et al.* 2014) with default settings, including standard Illumina adapters, and visually checked for quality using FastQC (Andrews 2010). Processed raw reads were *de novo* assembled using Minia v.2.0 (Chikhi and Rizk 2013) with the command line arguments, -kmer-size 121. K-mer size was optimized by iteratively assembling the genomes -kmer-size = 41–141 and the optimal k-mer size was selected based on assembly statistics (Chikhi and Rizk 2013). Comparative assembly statistics were compiled using Quast v 5.0.0 (Gurevich *et al.* 2013). For the average assembly, the total length was 1.75 Mbp (range 1.55:2.05 Mbp), with 25 total contigs (range 19:38). The average N50 was 327 kb (range 72.6:491 kb). The average L50 was 4 (2:8), and GC content was 36% (range 35.7%:36.8%) (Supplementary Table 1). Minia generated contig files were used for downstream analyses.

To assess assembly completeness (accuracy of assembled orthologs), we analyzed the genome assemblies for Benchmarking Universal Single-Copy Orthologs (BUSCOs), which are genes that are expected to be present in closely related bacteria. To do this, we used BUSCO v.4.1.3 (Simão *et al.* 2015) with the Lactobacillales database v.10. All 3 genomes contained at least 98% of expected BUSCOs, suggesting they were relatively complete.

### Phylogenetic analysis

**Fig. 1.**
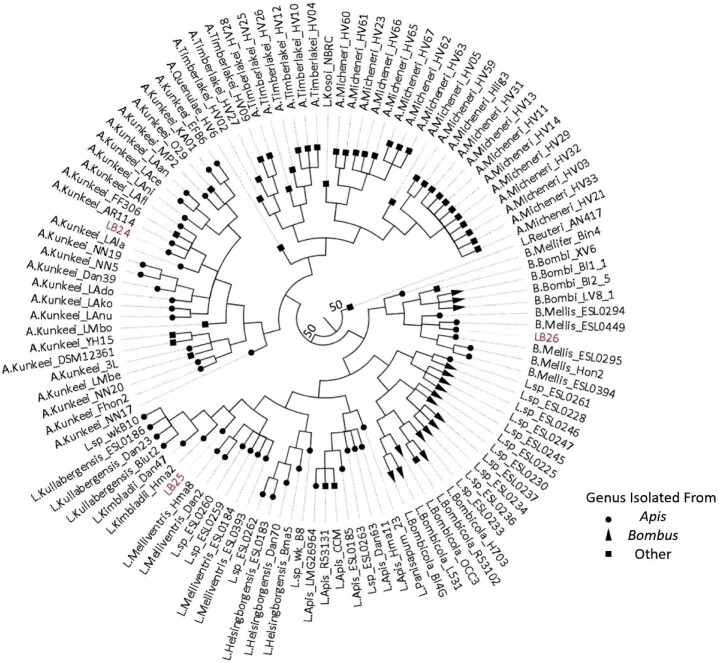
Phylogenetic tree of 110 bee-associated members of the Lactobacilliaceae family plus *L. reuteri* AN417 (outgroup). Tip shape is based on the host genus the bacteria was isolated from; circle (*Apis* = honey bee), triangle (*Bombus* = bumble bee), and square (other). Only bootstrap values below 100 are shown.

In addition to our 3 *Lactobacillus* isolates, 107 additional whole-genome sequences of all bee-associated members of the Lactobacillaceae family, as well as the genome of *Limosilactobacillus reuteri* AN417 [=*Lactobacillus reuteri* (Zheng *et al.* 2020)], were downloaded from Genbank for use in phylogenetic analysis (Supplementary Table 2). *Limosilactobacillus reuteri* AN417 was used as an outgroup for our bee-associated Lactobacillaceae tree (*N* = 111 genomes). All genomes were annotated using Prokka v.1.14.6 using the default databases (Seemann 2014). To generate a core gene alignment, PIRATE (Bayliss *et al.* 2019) was run using the general feature format files generated by Prokka. The resulting core alignment contained 359 genes. A Lactobacillaceae phylogenetic tree was reconstructed using this core genome alignment in RAxML HPC v.8.2.12 (Stamatakis 2014). RAxML was run using a random number seed for parsimony inferences, rapid bootstrapping with 1,000 replicates, and the GTRCAT nucleotide substitution model. The resulting consensus tree was rooted and converted to nexus format using Geneious prime v.2020.2.1 (Biomatters Ltd). The tip labels were aligned, and tip colors were changed to highlight the placement of our isolates. Bootstrap values below 100 were visualized using FigTree v.1.4.4 (Rambaut 2010) (Fig. 1).

### Comparative genomic analysis

**Fig. 2.**
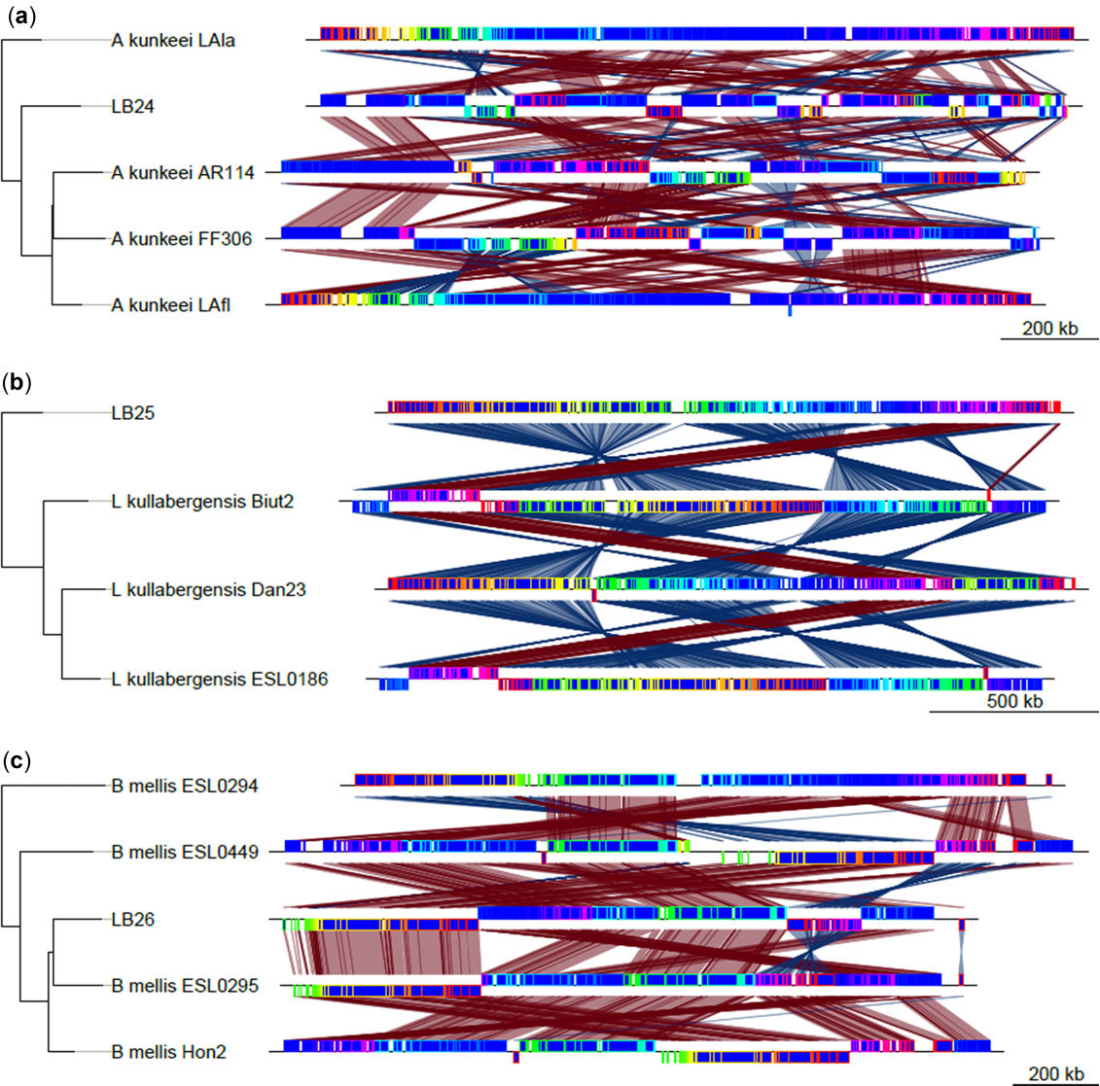
Mauve alignments of the 3 Lactobacillaceae isolates with closely related known species. Isolate LB24 aligned with 4 closely related *Apilactobacillus kunkeei* isolates (a). Isolate LB25 aligned with 3 closely related *Lactobacillus kullabergensis* isolates (b). Isolate LB26 aligned with 4 closely related *Bombilactobacillus mellis* isolates. Rearrangement of syntenic blocks are shown in red, and inversion of syntenic blocks are shown in blue.

**Fig. 3.**
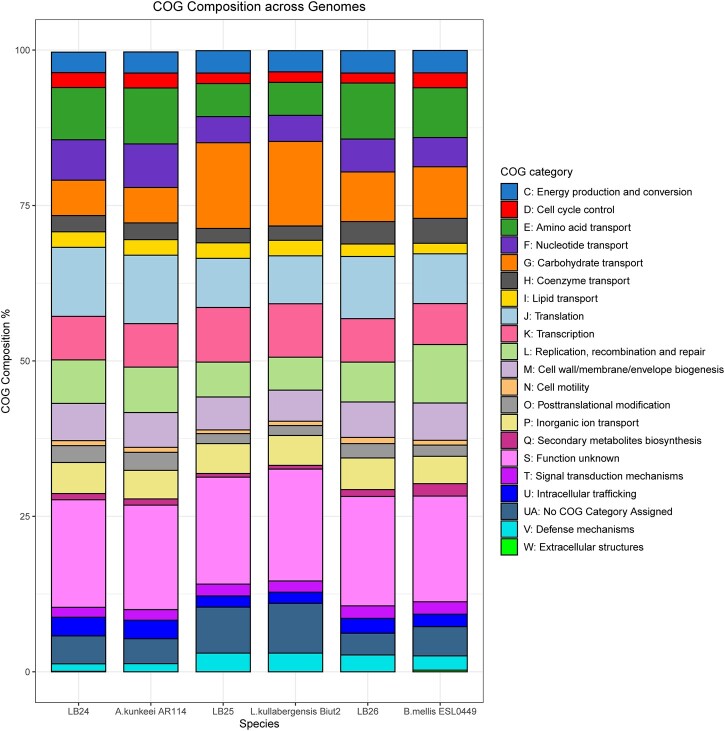
Stacked bar chart comparing the COG category composition between LB24, LB25, LB26, and their most closely related isolate: *Apilactobacillus kunkeei* AR114, *Lactobacillus kullabergensis* Biut2, and *Bombilactobacillus mellis* ESL0449, respectively. LB24 and *B. mellis* ESL0449 both contained genes that were assigned to COG category “W”; however, in both species that category represented <0.5% of the genes which makes it difficult to see on the figure. A more detailed description of each COG category can be found in Supplementary Table 3.

To visualize rearrangements within the genomes, Mauve build v.2.4.0 (Darling *et al.* 2004, 2010) was used. Our isolates, as well as a few of their closest relatives selected from the phylogenetic tree produced from all 111 genomes, were aligned using the default settings of progressive mauve. A phylogenetic tree was then added to these alignments in R v.4.0.5 (R Core Team 2021) using the genoPlotR package v.0.8.11 (Guy *et al.* 2010) (Fig. 2). To compare COG functional categories across genomes, our isolates, as well as the genomes of their closest relatives (*Apilatobacillus kunkeei* AR114, *Lactobacillus kullabergensis* Biut2 and *Bombilactobacillus mellis* ESL0449; Genbank accession numbers GCF_000830375.1, GCF_000967195.1, and GCF_013346905.1, respectively), were annotated using eggNOG-mapper v.2.0 with eggNOG database v.5.0 and the DIAMOND algorithm (Buchfink *et al.* 2015; Huerta-Cepas *et al.* 2017, 2019). The total number of genes per COG category were divided by the total number of genes within the genome. The resulting percentages were then plotted in R v.4.0.5 using ggplot2 v.3.3.5 (Wickham 2016) (Fig. 3). For the sake of brevity, the COG descriptions used in the legend of Fig. 3 have been truncated. The full COG descriptions are included in Supplementary Table 3.

### Prophage investigation

To check for the presence of prophage, the 3 genomes were analyzed with VirSorter2 v.2.2.2 (Guo *et al.* 2021). To reduce false positives and trim bacterial ends from potential prophage regions, potential regions scored above 0.9 by VirSorter2 were then analyzed with VIBRANT v.1.2.1, and those classified as viral were considered prophages (Kieft *et al.* 2020).

**Fig. 4.**
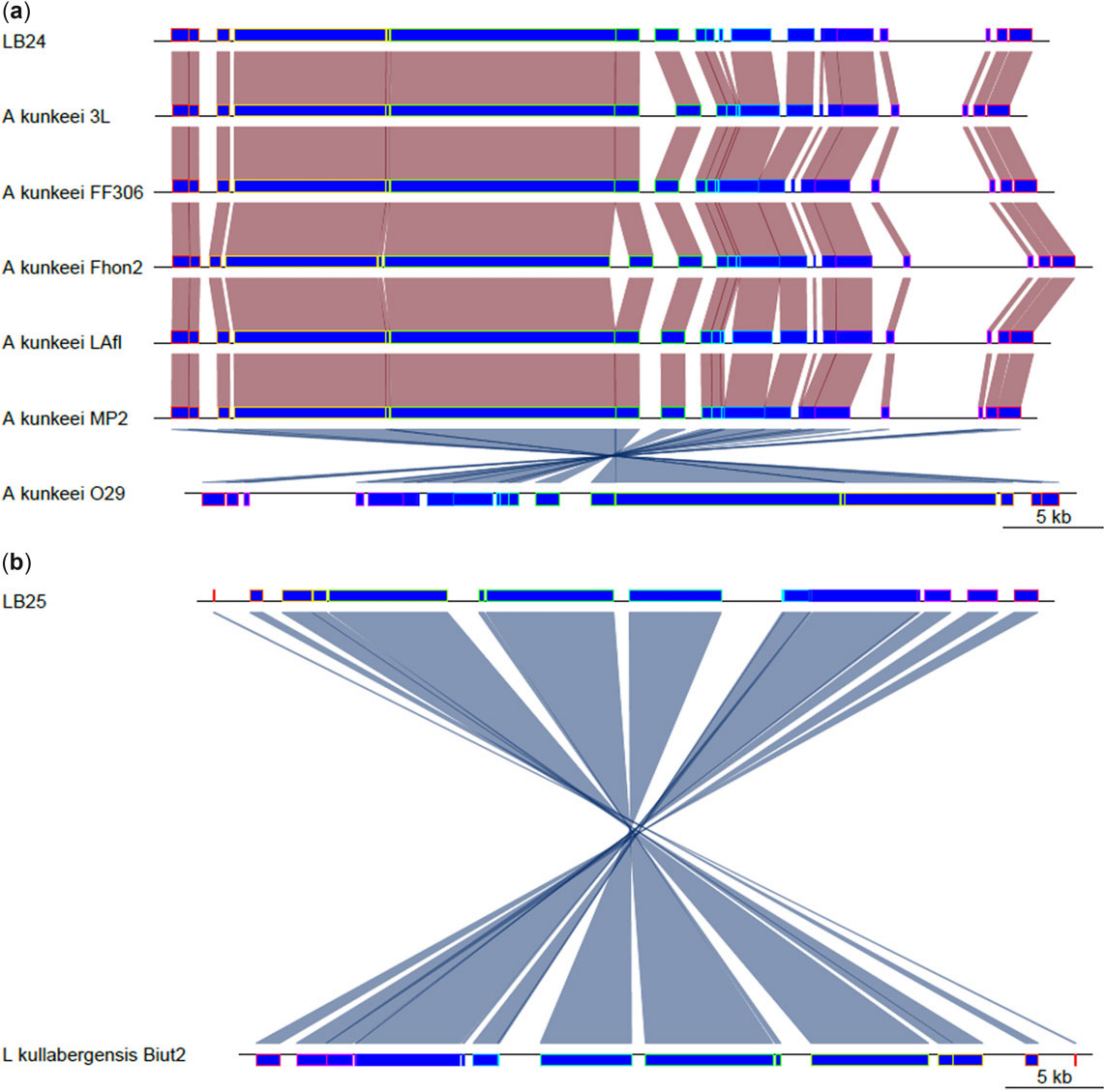
Mauve alignments of the most similar prophage(s) to those in LB24 and LB25 found in related species. Alignments shown share more than 80% of ANI to at least half of either LB24 or LB25’s prophage ORFs. Isolate LB24 with 6 *Apilactobacillus kunkeei* isolates (a). Isolate LB25 with one other *Lactobacillus kullabergensis* isolate (b). Prophage regions were predicted bioinformatically and therefore, this does not necessarily confirm the presence of active phages.

Additionally, a BLAST database was created using the 2 phages found in LB24 and LB25 and run against all 107 bee-associated members of the Lactobacillaceae family used in this study. Bacterial genomes with *E* scores of 0 were then analyzed with VirSorter2 and VIBRANT. The score threshold for VirsSorter2 was relaxed to 0.5 to include putative prophage regions in the reference genomes that may be partially degraded or cryptic but were still confirmed and trimmed with VIBRANT. The open reading frames (ORFs) of each putative prophage were identified with Prodigal (Hyatt *et al.* 2010) and average nucleic acid (ANI) similarity between phages was calculated using BLAST. Prophage from reference genomes were also aligned to the LB24 and LB25 prophage regions using progressive Mauve and visualized in R v.4.0.5 (R Core Team 2021) using the genoPlotR package v.0.8.11 (Guy *et al.* 2010) (Fig. 4).

## Results and discussion

Using the nucleotide sequence of the 16S rRNA gene extracted from the whole-genome sequences, we identified LB24 as a novel strain of *Apilactobacillus kunkeei*, LB25 as *Lactobacillus kullabergensis* and LB26 as *Bombilactobacillus mellis* (Zheng *et al.* 2020). The core genome of the 110 bee-associated members of the Lactobacillaceae family contained 359 genes, as did the *Limosilactobacillus reuteri* strain AN417 isolated from domestic pigs that served as an outgroup in our phylogeny. The phylogenetic tree created using the core genome alignment confirmed the initial identifications of LB24 (*A. kunkeei*) and LB26 (*B. mellis*) (Fig. 1). However, in this tree, LB25 was placed at the root of *L. kullabergenis* and *L. kimbladii*, suggesting it was likely to be closely related to both of these, but could not be unambiguously placed as one of those 2 species (Fig. 1). Because of this unclear phylogenetic placement, we used sequence homology to determine the most closely related species to LB25 for subsequent comparative analyses. In this case, 30 full-length genes were aligned to the NCBI nr database using BLAST. Of these genes, 100% retrieved a strain of *Lactobacillus kullabergensis* as the best hit, so we used that species for comparative analyses for LB25.

*Apilactobacillus kunkeei* [=*Lactobacillus kunkeei* (Zheng *et al.* 2020)] is a fructophilic lactic acid bacterium, with previous isolates found within wine, flowers, honey, and honey bees (Endo *et al.* 2012; Neveling *et al.* 2012). The assembled *A. kunkeei* isolate (LB24) has a similar genome size of 1,558,246 bp (1.407–1.634 Mb) and GC content, 37% (35.47–36.57%) compared with the 59 published *A. kunkeei* genomes. We identified a lower number of predicted coding sequences (1,294 compared with 1,338–1,384) and similar number of tRNA genes (62 compared with 31–65), as 3 other newly described species isolated from honey bee guts (Crovadore *et al.* 2021), which may result from assembly level differences. Unlike Crovadore *et al.* (2021), we identified a prophage region in the LB24 assembly, consistent with other *A. kunkeei* MP2 assemblies (Asenjo *et al.* 2016). Additionally, the prophage identified in LB24 shares approximately half its genes, with high nucleotide similarity, to prophage regions found in MP2 and 5 other strain assemblies of *A. kunkeei*. Functional analysis of the LB24 assembly revealed that 1,229 of the 1,291 predicted coding sequences were assigned COG categories, which along with BUSCO scores supports the integrity of this assembly. Categorization was largely similar between the LB24 assembly and a reference strain (Fig. 3). Investigating the genes unique to LB24 we identified S (Function unknown; 23% of genes) as the largest, with L (Replication, recombination and repair; 12% of genes) as the second most abundant of the assigned categories. Many of the genes in these 2 groups (18% and 22%, respectively) included some with annotations related to phage (e.g. ybl78: conserved phage C-terminus, and sip: phage integrase family). While the functional importance of this categorical expansion, and prophage presence in LB24 needs to be tested, in studies of human commensal *Lactobacillus johnsonii*, these genes have been associated with genome scale rearrangements that have facilitated host specific adaptation (Guinane *et al.* 2011). While a direct connection remains to be established, consistent with this concept, we identified rearrangements between this assembly and reference strains (Fig. 2a).

*Lactobacillus kullabergensis* has been isolated from both the honey stomach and gut of honey bees (Olofsson *et al.* 2014). The assembly of LB25, putatively, *L. kullabergensis*, had a genome size of 2,052,702 bp (2.019–2.118 Mb) and the same GC content 36%, as seen in the 6 published genomes on NCBI. The LB25 assembly has marginally lower predicted coding sequences (1,833 compared with 1,844) as well as number of tRNA genes (53 compared with 50) compared with the sequence used by Ellegaard *et al.* (2015) in their investigation of intraphylotype diversity in Lactobacilli. As with our *A. kunkeei* isolate (LB24), a prophage region was identified within our *L. kullabergensis* isolate. The prophage in our isolate shared some similarity to a prophage identified in the *L. kullabergensis* Biut2 isolate, and similarity to putative prophage in *L. melliventris* isolates, albeit with much weaker support. Functional analysis revealed that 1,686 of the 1,827 predicted coding sequences from the LB25 assembly were assigned COG categories. As with the LB24 assembly, categorization was largely similar to that of the reference strain (Fig. 3). Investigating genes that were unique to the LB25 assembly, we found an increased number of unique genes associated with carbohydrate transport, with 34% of the uniquely identified genes falling in this category. This is consistent with other studies of honey bee isolated *L. kullabergensis* strains (Ellegaard *et al.* 2015) and it has been associated with increased metabolic flexibility among host-associated *Lactobacillus* spp. (Barrangou *et al.* 2006). When LB25 is aligned with the closely related *L. kullabergensis* Biut2, the inversion of several syntenic blocks is observed, and these are also seen when *L. kullabergensis* Biut2 is aligned with the 2 other known *L. kullabergensis* strains (Fig. 2b). Together these suggest potential, testable, mechanisms through which LB25 and other *L. kullabergensis* strains may adapt to diverse hosts.

*Bombilactobacillus mellis* [=*Lactobacillus mellis* (Zheng *et al.* 2020)] has been isolated from honey, bee bread, pollen, the honey stomach, gut, and digestive tract of *A. mellifera* and other *Apis* species (Olofsson *et al.* 2014). The LB26 *B. mellis* assembled with a genome size of 1,644,951 bp (1.684–1.811 MB) and a GC content of 36%, similar to the 8 published genomes on NCBI. This assembly had a lower number of predicted coding sequences (1,415 compared with 1,572), and fewer tRNA genes (38 compared with 53) when compared with the strain sequenced by Ellegaard *et al.* (2015) in their investigation of intraphylotype diversity in Lactobacilli. Functional analysis of the LB26 assembly assigned 1,359 of the 1,411 predicted coding sequences to COG categories. Once again, categorization was similar to that of the reference strain (Fig. 3). Analysis of the unique genes revealed a dominance of genes with unknown function, similar to the *A. kunkeei* isolate LB24. In contrast, the number of annotations associated with differences in transcription or transcriptional regulation were markedly greater (17% vs 7%) than those found in common between LB26 and the reference strain. While beyond the scope of the present study, in other lactobacilli, changes in the regulation of transcription have been associated with increased host-specificity (Goh *et al.* 2021). Unlike the *A. kunkeei* and *L. kullabergensis* isolates in our study, no prophage regions were identified within the *B. mellis* isolate. While the lack of phage in this isolate is rare, as Pei *et al.* (2021) found 99.8% of Lactobacillaceae genomes investigated had prophage regions, no *B. mellis* strains were included in that study. For LB26 and the 4 most closely related strains of *B. mellis* (ESL0294, ESL0449, ESL0295, and Hon2), the Mauve alignment suggested many of the syntenic blocks had undergone rearrangements among the genomes (Fig. 2c).

Intriguingly, we identified predicted prophage regions in 2, LB24 and LB25, of the 3 assembled genomes. No prophage was predicted in LB26. In LB24, a single predicted prophage region was identified with a probability >0.9 using VirSorter2 (dsDNA phage, 89,958 bp), which was trimmed to 56,656 bp by VIBRANT. In LB25, there was also 1 predicted prophage region identified with a probability >0.9 (dsDNA phage, 43,795 bp), which was not trimmed by VIBRANT. It is possible that the predicted prophage in LB24 prophage may be partially degraded or possess novel genes, disrupting the analysis software. Conversely, the prophage predicted in the LB25 prophage may be more intact and/or have genes that are more recognizably phage-like. The comparative alignments of prophage regions among closely related strains are consistent with general genomic rearrangements (Fig. 4).

While it remains to be determined that these predicted prophage regions are inducible, we identified shared homology with these regions and prophage identified in other bee-associated Lactobacillaceae. The putative prophage of LB24 shared high ANI across aligned ORFs with 12 other predicted phage regions, appearing most similar to prophages found in 6 isolates of *A. kunkeei* (LAfl, FF306, 3L, Fhon2, O29, MP2), with 92.23–96.80% ANI across 55.3–81.6% of the LB24 assembled phage ORFs (Fig. 4a and Supplementary Table 4). Phage from other isolates of *A. kunkeei* (Laan, Dan39) and other species like *Apilactobacillus quenuiae* (HV6) and *Apilactobacillus micheneri* (HV05, HV61, HV60) shared between 1 and 3 ORFs (1.3–4.0% of LB24’s phage ORFs) with ANI ranging from 73.9 to 93.86% (Supplementary Table 4). This may indicate that a high amount of horizontal gene transfer occurred between these prophages, resulting in mosaic genomes (Vale *et al.* 2017; Moura de Sousa *et al.* 2021). The most similar prophage region to that found in LB25 prophage was identified in *L. kullabergensis* Biut2 with an ANI of 88.0% across 53.3% of the LB25 assembled phage ORFs (Fig. 4b and Supplementary Table 4). Additional less confident matches to the phage occurred in 4 isolates of *L. melliventris* (ESL0184, ESL0393, Hma8, Dan2), 2 isolates of *L. helsingborgensis* (ESL0183, Dan70) and in 1 *Lactobacillus apis* isolate (Dan63), with a range of 84.8–90.5% ANI across 20.0–28.3% of the assembled phage ORFs (Supplementary Table 4). Finally, LB25’s phage also matched slightly with phage found in 3 isolates of *Lactobacillus bombicola* (BI4G, L531, OCC3), each sharing 3 ORFs (5.0% of LB25’s ORFs) with ANI of 76.7–76.9% (Supplementary Table 4). Additionally, the LB25 prophage region shared similarities with prophage regions found in 11 unidentified species of Lactobacillaceae (wkB10, ESL0263, ESL0262, ESL0261, ESL0237, ESL236, ESL0234, ESL0233, ESL0230, ESL0228, and ESL0225) with 76.5–90.5% ANI across 1.7–21.7% of LB25’s ORFs (Supplementary Table 4).

This study presents the assembly and annotation of 3 novel strains of Lactobacillaceae isolated from the honey bee gut. Phylogenetic placement of the strains was well supported, with additional support coming from syntenic and functional analyses. While we have a number of insights on the probiotic nature of this family in the human gut (Zhang *et al.* 2018), their role in the honey bee gut is just emerging. These complete genome assemblies can serve as references for assembling additional strains, and gleaning further genomic and mechanistic insights among members of this group that form close associations with honey bees.

## Supplementary Material

jkac286_Supplementary_Material

jkac286_Supplementary_Table_S1

jkac286_Supplementary_Table_S2

jkac286_Supplementary_Table_S3

jkac286_Supplementary_Table_S4

## Data Availability

Genome assemblies and raw data can be found here: http://www.ncbi.nlm.nih.gov/bioproject/866146, but note that these assemblies have been cleaned of any contigs <200 bp by NCBI requirements. Full assemblies, processing scripts, and associated metadata can be obtained from, https://doi.org/10.7294/19524946. File LB24_final_contigs.fasta contains the complete final contig list of LB24. File LB25_final_contigs.fasta contains the complete final contig list of LB25. File LB26_final_contigs.fasta contains the complete final contig list of LB26. File lactobacillus_MS_metadata_scripts_readme.docx contains all scripts used in the metadata analysis in a word document. File 09_scripts.zip contains all scripts used in the metadata analysis. Supplemental material is available at G3 online.
