# Supplementary material for: Comparative genomics of Lactobacillaceae from the gut of honey bees, Apis mellifera, from the Eastern United States
Source: G3 (Bethesda). 2022 Nov 4;12(12):jkac286. doi: 10.1093/g3journal/jkac286 (PMC9713430; doi:10.1093/g3journal/jkac286)
Supplement: jkac286_Supplementary_Material [file jkac286_supplementary_material.docx]

Comparative genomics of Lactobacillaceae from the gut of honey bees, *Apis mellifera*, from the Eastern US.

Emma L. Bradford^*1^, Noah Wax^*^, Emma K. Bueren^*^, Jenifer B. Walke^†^, Richard Fell^‡^, Lisa K. Belden^*^, David C. Haak^§1^

^*^Department of Biological Sciences, Virginia Tech, Blacksburg, Virginia, 24061, USA

^†^Department of Biology, Eastern Washington University, Cheney, Washington, 99004, USA

^‡^Department of Entomology, Virginia Tech, Blacksburg, Virginia, 24061, USA

^§^School of Plant and Environmental Sciences, Virginia Tech, Blacksburg, Virginia, 24061, USA

^1^Corresponding author: Emma L. Bradford, [ebradford@vt.edu](mailto:ebradford@vt.edu), David C. Haak, [dhaak@vt.edu](mailto:dhaak@vt.edu)

Running title: 3 honey bee *Lactobacillus* isolates

Key words: *Lactobacillus*, *Apilactobacillus*, *Bombilactobacillus*, whole genome, honey bee microbiome, gut microbiome, prophage, bacteriophage

Table S1: Quast assembly statistics of Lactobacillaceae isolates LB24, LB25 and LB26.

Table S2: Genbank accession numbers of bee associated Lactobacillaceae species.

Table S3: Detailed description of COG Functional Categories.

Table S4: Average nucleotide identity of shared ORF of putative LB24 or LB25 prophage with prophage identified in reference of bee associated Lactobacillaceae species.
