# Supplementary material for: Comparative genomics of Lactobacillaceae from the gut of honey bees, Apis mellifera, from the Eastern United States
Source: G3 (Bethesda). 2022 Nov 4;12(12):jkac286. doi: 10.1093/g3journal/jkac286 (PMC9713430; doi:10.1093/g3journal/jkac286)
Supplement: jkac286_Supplementary_Table_S1 [file jkac286_supplementary_table_s1.docx]

| Assembly | LB24_gapclose_final_scaffolds | LB25_final_contigs | LB26_final_contigs |
| --- | --- | --- | --- |
| # contigs (>= 0 bp) | 38 | 19 | 19 |
| # contigs (>= 1000 bp) | 38 | 9 | 10 |
| # contigs (>= 5000 bp) | 31 | 6 | 8 |
| # contigs (>= 10000 bp) | 29 | 6 | 8 |
| # contigs (>= 25000 bp) | 23 | 5 | 7 |
| # contigs (>= 50000 bp) | 10 | 5 | 5 |
| Total length (>= 0 bp) | 1558246 | 2052702 | 1644951 |
| Total length (>= 1000 bp) | 1558246 | 2050538 | 1643009 |
| Total length (>= 5000 bp) | 1541762 | 2041434 | 1638600 |
| Total length (>= 10000 bp) | 1526382 | 2041434 | 1638600 |
| Total length (>= 25000 bp) | 1420110 | 2031090 | 1627637 |
| Total length (>= 50000 bp) | 951910 | 2031090 | 1555077 |
| # contigs | 38 | 19 | 19 |
| Largest contig | 177040 | 901259 | 471337 |
| Total length | 1558246 | 2051131 | 1643009 |
| GC (%) | 36.83 | 35.77 | 36.49 |
| N50 | 72673 | 491938 | 416952 |
| N90 | 25700 | 266758 | 171166 |
| L50 | 8 | 2 | 2 |
| L90 | 23 | 4 | 5 |
| # N's per 100 kbp | 21.95 | 0 | 0 |

Quast assembly statistics of *Lactobacillus* isolates LB24, LB25 and LB26.
