# Supplementary material for: Comparative genomics of Lactobacillaceae from the gut of honey bees, Apis mellifera, from the Eastern United States
Source: G3 (Bethesda). 2022 Nov 4;12(12):jkac286. doi: 10.1093/g3journal/jkac286 (PMC9713430; doi:10.1093/g3journal/jkac286)
Supplement: jkac286_Supplementary_Table_S2 [file jkac286_supplementary_table_s2.docx]

Genbank accession numbers of bee associated *lactobacillus* species.

| FNA File Name | RefSeq.assembly.accession number |
| --- | --- |
| Apilactobacillus_Kunkeei_3L.fna | GCF_006493615.1 |
| Apilactobacillus_Kunkeei_AR114.fna | GCF_000830375.1 |
| Apilactobacillus_Kunkeei_Dan39.fna | GCF_014323645.1 |
| Apilalctobacillus_Kunkeei_DSM12361.fna | GCF_001433825.1 |
| Apilactobacillus_Kunkeei_EFB6.fna | GCF_000687335.1 |
| Apilactobacillus_Kunkeei_FF306.fna | GCF_001949975.1 |
| Apilactobacillus_Kunkeei_Fhon2.fna | GCF_001281165.1 |
| Apilactobacillus_Kunkeei_KA01.fna | GCF_006493415.1 |
| Apilactobacillus_Kunkeei_LAan.fna | GCF_001281225.1 |
| Apilactobacillus_Kunkeei_LAce.fna | GCF_001421115.1 |
| Apilactobacillus_Kunkeei_LAdo.fna | GCF_001308205.1 |
| Apilactobacillus_Kunkeei_LAfl.fna | GCF_001421135.1 |
| Apilactobacillus_Kunkeei_LAko.fna | GCF_001281205.1 |
| Apilactobacillus_Kunkeei_LAla.fna | GCF_001281215.1 |
| Apilactobacillus_Kunkeei_LAni.fna | GCF_001281285.1 |
| Apilactobacillus_Kunkeei_LAnu.fna | GCF_001308215.1 |
| Apilactobacillus_Kunkeei_LMbe.fna | GCF_001308185.1 |
| Apilactobacillus_Kunkeei_LMbo.fna | GCF_001308195.1 |
| Apilactobacillus_Kunkeei_MP2_complete.fna | GCF_001314945.1 |
| Apilactobacillus_Kunkeei_NN17.fna | GCF_005930975.1 |
| Apilactobacillus_Kunkeei_NN19.fna | GCF_005930925.1 |
| Apilactobacillus_Kunkeei_NN20.fna | GCF_005930935.1 |
| Apilactobacillus_Kunkeei_NN5.fna | GCF_005930915.1 |
| Apilactobacillus_Kunkeei_O29.fna | GCF_009910775.1 |
| Apilactobacillus_Kunkeei_YH15.fna | GCF_001281265.1 |
| Apilactobacillus_Micheneri_Hlig3.fna | GCF_002993975.1 |
| Apilactobacillus_Micheneri_HV03.fna | GCF_006493495.1 |
| Apilactobacillus Micheneri_HV05.fna | GCF_006493295.1 |
| Apilactobacillus Micheneri_HV11.fna | GCF_006493545.1 |
| Apilactobacillus Micheneri_HV13.fna | GCF_006493185.1 |
| Apilactobacillus Micheneri_HV14.fna | GCF_006493225.1 |
| Apilactobacillus Micheneri_HV21.fna | GCF_006493275.1 |
| Apilactobacillus Micheneri_HV23.fna | GCF_006493625.1 |
| Apilactobacillus Micheneri_HV29.fna | GCF_006493235.1 |
| Apilactobacillus Micheneri_HV31.fna | GCF_006493515.1 |
| Apilactobacillus_Micheneri_HV32.fna | GCF_006493195.1 |
| Apilactobacillus_Micheneri_HV33.fna | GCF_006493445.1 |
| Apilactobacillus_Micheneri_HV59.fna | GCF_006493525.1 |
| Apilactobacillus_Micheneri_HV60.fna | GCF_006493325.1 |
| Apilactobacillus_Micheneri_HV61.fna | GCF_006493375.1 |
| Apilactobacillus_Micheneri_HV62.fna | GCF_006493305.1 |
| Apilactobacillus_Micheneri_HV63.fna | GCF_006493315.1 |
| Apilactobacillus_Micheneri_HV65.fna | GCF_006493595.1 |
| Apilactobacillus_Micheneri_HV66.fna | GCF_006493385.1 |
| Apilactobacillus_Micheneri_HV67.fna | GCF_006493565.1 |
| Apilactobacillus_Quenuiae_HV6.fna | GCF_002994005.1 |
| Apilactobacillus_Timberlakei_HV02.fna | GCF_006493435.1 |
| Apilactobacillus_Timberlakei_HV04.fna | GCF_006493055.1 |
| Apilactobacillus_Timberlakei_HV09.fna | GCF_006493175.1 |
| Apilactobacillus_Timberlakei_HV10.fna | GCF_006493095.1 |
| Apilactobacillus_Timberlakei_HV12.fna | GCF_002993965.1 |
| Apilactobacillus_Timberlakei_HV25.fna | GCF_006493105.1 |
| Apilactobacillus_Timberlakei_HV26.fna | GCF_006493425.1 |
| Apilactobacillus_Timberlakei_HV27.fna | GCF_006493155.1 |
| Apilactobacillus_Timberlakei_HV28.fna | GCF_006493125.1 |
| Bombilactobacillus_Bombi_BI1.1.fna | GCF_003515805.1 |
| Bombilactobacillus_Bombi_BI2.5.fna | GCF_003522965.1 |
| Bombilactobacillus_Bombi_LV8.1.fna | GCF_003515755.1 |
| Bombilactobacillus_Bombi_XV6.fna | GCF_013607485.1 |
| Bombilactobacillus_Mellifer_Bin4.fna | GCF_000970795.1 |
| Bombilactobacillus_Mellis_ESL0294.fna | GCF_013346925.1 |
| Bombilactobacillus_Mellis_ESL0295.fna | GCF_013345055.1 |
| Bombilactobacillus_Mellis_ESL0394.fna | GCF_013347085.1 |
| Bombilactobacillus_Mellis_ESL0449.fna | GCF_013346905.1 |
| Bombilactobacillus_Mellis_Hon2.fna | GCF_000967245.1 |
| Lactobacillus_Apis_CCM8403.fna | GCF_014635365.1 |
| Lactobacillus_Apis_Dan63.fna | GCF_014324145.1 |
| Lactobacillus_Apis_ESL0185.fna | GCF_003150935.1 |
| Lactobacillus_Apis_Hma11.fna | GCF_000970735.1 |
| Lactobacillus_Apis_LMG26964.fna | GCF_002837055.1 |
| Lactobacillus_Apis_R53131.fna | GCF_900094785.1 |
| Lactobacillus_Bombicola_BI4G.fna | GCF_003515845.1 |
| Lactobacillus_Bombicola_H703.fna | GCF_002916915.1 |
| Lactobacillus_Bombicola_L531.fna | GCF_003515825.1 |
| Lactobacillus_Bombicola_OCC3.fna | GCF_003515855.1 |
| Lactobacillus_Bombicola_R53102.fna | GCF_900112665.1 |
| Lactobacillus_Helsingborgensis_Bma5.fna | GCF_000970855.1 |
| Lactobacillus_Helsingborgensis_Dan70.fna | GCF_014324155.1 |
| Lactobacillus_Helsingborgensis_ESL0183.fna | GCF_003173695.1 |
| Lactobacillus_Kimbladii_Dan47.fna | GCF_014323605.1 |
| Lactobacillus_Kimbladii_Hma2.fna | GCF_000970755.1 |
| Lactobacillus_Kosoi_NBRC113063.fna | GCF_003112615.1 |
| Lactobacillus_Kullabergensis_Biut2.fna | GCF_000967195.1 |
| Lactobacillus_Kullabergensis_Dan23.fna | GCF_014324215.1 |
| Lactobacillus_Kullabergensis_ESL0186.fna | GCF_003151025.1 |
| Lactobacillus_Melliventris_Dan2.fna | GCF_014324185.1 |
| Lactobacillus_Melliventris_ESL0184.fna | GCF_003202825.1 |
| Lactobacillus_Melliventris_ESL0393.fna | GCF_013346935.1 |
| Lactobacillus_Melliventris_Hma8.fna | GCF_000970775.1 |
| Lactobacillus_Panisapium_23.fna | GCF_002916935.1 |
| Lactobacillus_Reuteri_AN417.fna | GCF_013348825.1 |
| Lactobacillus_sp_ESL0225.fna | GCF_003692905.1 |
| Lactobacillus_sp_ESL0228 | GCF_003692825.1 |
| Lactobacillus_sp_ESL0230 | GCF_003692805.1 |
| Lactobacillus_sp_ESL0233 | GCF_003692995.1 |
| Lactobacillus_sp_ESL0234 | GCF_003692885.1 |
| Lactobacillus_sp_ESL0236 | GCF_003692775.1 |
| Lactobacillus_sp_ESL0237 | GCF_003692985.1 |
| Lactobacillus_sp_ESL0245 | GCF_003692865.1 |
| Lactobacillus_sp_ESL0246 | GCF_003692965.1 |
| Lactobacillus_sp_ESL0247 | GCF_003692755.1 |
| Lactobacillus_sp_ESL0259 | GCF_003693045.1 |
| Lactobacillus_sp_ESL0260 | GCF_003692935.1 |
| Lactobacillus_sp_ESL0261 | GCF_003693025.1 |
| Lactobacillus_sp_ESL0262 | GCF_003692925.1 |
| Lactobacillus_sp_ESL0263 | GCF_003692845.1 |
| Lactobacillus_sp_wkB10 | GCF_000760615.1 |
| Lactobacillus_sp_wkB8 | GCF_000761135.1 |
