# Supplementary material for: Comparative genomics of Lactobacillaceae from the gut of honey bees, Apis mellifera, from the Eastern United States
Source: G3 (Bethesda). 2022 Nov 4;12(12):jkac286. doi: 10.1093/g3journal/jkac286 (PMC9713430; doi:10.1093/g3journal/jkac286)
Supplement: jkac286_Supplementary_Table_S3 [file jkac286_supplementary_table_s3.docx]

Detailed description of COG Functional Categories.

| COG Category | Full Description |
| --- | --- |
| C | Energy production and conversion |
| D | Cell cycle control, cell division, chromosome partitioning |
| E | Amino acid transport and metabolism |
| F | Nucleotide transport and metabolism |
| G | Carbohydrate transport and metabolism |
| H | Coenzyme transport and metabolism |
| I | Lipid transport and metabolism |
| J | Translation, ribosomal structure and biogenesis |
| K | Transcription |
| L | Replication, recombination and repair |
| M | Cell wall/membrane/envelope biogenesis |
| N | Cell motility |
| O | Posttranslational modification, protein turnover, chaperones |
| P | Inorganic ion transport and metabolism |
| Q | Secondary metabolites biosynthesis, transport and catabolism |
| S | Function unknown |
| T | Signal transduction mechanisms |
| U | Intracellular trafficking, secretion, and vesicular transport |
| UA | No COG Category Assigned |
| V | Defense mechanisms |
| W | Extracellular structures |
