# Supplementary material for: Comparative genomics of Lactobacillaceae from the gut of honey bees, Apis mellifera, from the Eastern United States
Source: G3 (Bethesda). 2022 Nov 4;12(12):jkac286. doi: 10.1093/g3journal/jkac286 (PMC9713430; doi:10.1093/g3journal/jkac286)
Supplement: jkac286_Supplementary_Table_S4 [file jkac286_supplementary_table_s4.docx]

Average nucleotide identity of shared ORF of putative LB24 or LB25 prophage with prophage identified in reference of bee associated Lactobacillaceae species.

| Phage1 (P1) | Phage2 (P2) | ANI | P1 ORFs | Hits to P1 | Percent of LB24 or LB25’s Hits to Reference Phage |
| --- | --- | --- | --- | --- | --- |
| LB24_Phage | LaKunkeei_LAfl_10_full_fragment_1 | 96.8019032 | 76 | 62 | 81.58 |
| LB24_Phage | LaKunkeei_FF306_1_0_partial_fragment_1 | 94.6324074 | 76 | 54 | 71.05 |
| LB24_Phage | LaKunkeei_3L_28_full | 96.6682692 | 76 | 52 | 68.42 |
| LB24_Phage | LaKunkeei_Fhon2_2_0_partial_fragment_1 | 93.4921489 | 76 | 47 | 61.84 |
| LB24_Phage | LaKunkeei_O29_2_0_partial_fragment_1 | 93.0145227 | 76 | 44 | 57.89 |
| LB24_Phage | LaKunkeei_MP2_complete_1_0_partial_fragment_1 | 92.2268095 | 76 | 42 | 55.26 |
| LB24_Phage | LaKunkeei_Dan39_3_full | 84.4443333 | 76 | 3 | 3.95 |
| LB24_Phage | ApMicheneri_HV05_1_0_partial_fragment_1 | 76.9813333 | 76 | 3 | 3.95 |
| LB24_Phage | LaKunkeei_LAan_10_0_partial_fragment_1 | 96.6245 | 76 | 2 | 2.63 |
| LB24_Phage | ApQuenuiae_HV6_1_0_partial_fragment_1 | 77.4965 | 76 | 2 | 2.63 |
| LB24_Phage | ApMicheneri_HV61_4_full | 73.881 | 76 | 1 | 1.32 |
| LB24_Phage | ApMicheneri_HV60_39_full | 73.881 | 76 | 1 | 1.32 |
| LB25_Phage | LaKullabergensis_Biut2_2_0_partial_fragment_1 | 87.9686875 | 60 | 32 | 53.33 |
| LB25_Phage | LaMelliventris_ESL0184_2_1_partial_fragment_1 | 90.5275882 | 60 | 17 | 28.33 |
| LB25_Phage | LaMelliventris_ESL0184_2_0_partial_fragment_1 | 84.758125 | 60 | 16 | 26.67 |
| LB25_Phage | LaMelliventris_Hma8_5_0_partial_fragment_1 | 90.2524375 | 60 | 16 | 26.67 |
| LB25_Phage | LaMelliventris_ESL0393_13_full | 90.1759333 | 60 | 15 | 25 |
| LB25_Phage | LaMelliventris_Dan2_2_full | 90.1759333 | 60 | 15 | 25 |
| LB25_Phage | LaHelsingborgensis_ESL0183_1_0_partial_fragment_1 | 90.4916154 | 60 | 13 | 21.67 |
| LB25_Phage | LaApis_Dan63_9_0_partial_fragment_1 | 84.5412308 | 60 | 13 | 21.67 |
| LB25_Phage | L_sp_ESL0262_6\|\|0_partial_fragment_1 | 90.4971538 | 60 | 13 | 21.67 |
| LB25_Phage | LaHelsingborgensis_Dan70_10_0_partial_fragment_1 | 88.93175 | 60 | 12 | 20 |
| LB25_Phage | L_sp_ESL0228_7\|\|0_partial_fragment_1 | 85.33275 | 60 | 8 | 13.33 |
| LB25_Phage | L_sp_ESL0261_7\|\|0_partial_fragment_1 | 85.33275 | 60 | 8 | 13.33 |
| LB25_Phage | L_sp_wkB10_7\|\|full_fragment_1 | 87.006125 | 60 | 8 | 13.33 |
| LB25_Phage | L_sp_ESL0263_4\|\|0_partial_fragment_1 | 82.9315 | 60 | 8 | 13.33 |
| LB25_Phage | L_sp_ESL0233_7\|\|0_partial_fragment_1 | 78.841 | 60 | 7 | 11.67 |
| LB25_Phage | L_sp_ESL0225_7\|\|0_partial_fragment_1 | 81.6368 | 60 | 5 | 8.33 |
| LB25_Phage | L_sp_ESL0236_8\|\|0_partial | 77.416 | 60 | 4 | 6.67 |
| LB25_Phage | L_sp_ESL0261_8\|\|0_partial_fragment_1 | 76.519 | 60 | 4 | 6.67 |
| LB25_Phage | L_sp_ESL0237_8\|\|0_partial | 77.416 | 60 | 4 | 6.67 |
| LB25_Phage | L_sp_ESL0228_8\|\|0_partial_fragment_1 | 76.514 | 60 | 4 | 6.67 |
| LB25_Phage | L_sp_ESL0234_8\|\|0_partial | 77.422 | 60 | 4 | 6.67 |
| LB25_Phage | L_sp_ESL0236_16\|\|full | 85.464 | 60 | 3 | 5 |
| LB25_Phage | LaBombicola_L531_17_0_partial_fragment_1 | 76.9453333 | 60 | 3 | 5 |
| LB25_Phage | L_sp_ESL0237_16\|\|full | 85.464 | 60 | 3 | 5 |
| LB25_Phage | LaBombicola_OCC3_3_full | 76.7436667 | 60 | 3 | 5 |
| LB25_Phage | LaBombicola_BI4G_1_0_partial_fragment_1 | 76.923 | 60 | 3 | 5 |
| LB25_Phage | L_sp_ESL0234_16\|\|full | 85.464 | 60 | 3 | 5 |
| LB25_Phage | L_sp_ESL0230_6\|\|0_partial_fragment_1 | 80.726 | 60 | 1 | 1.67 |
| LB25_Phage | L_sp_ESL0236_17\|\|full | 81.265 | 60 | 1 | 1.67 |
| LB25_Phage | L_sp_ESL0234_18\|\|full | 81.265 | 60 | 1 | 1.67 |
| LB25_Phage | L_sp_ESL0237_17\|\|full | 81.265 | 60 | 1 | 1.67 |
